# Deep-learning for automated detection of MSU deposits on DECT: evaluating impact on efficiency and reader confidence

**DOI:** 10.3389/fradi.2024.1330399

**Published:** 2024-02-19

**Authors:** Shahriar Faghani, Soham Patel, Nicholas G. Rhodes, Garret M. Powell, Francis I. Baffour, Mana Moassefi, Katrina N. Glazebrook, Bradley J. Erickson, Christin A. Tiegs-Heiden

**Affiliations:** ^1^ Artificial Intelligence Laboratory, Department of Radiology, Mayo Clinic, Rochester, MN, United States; ^2^ Department of Radiology, Mayo Clinic, Rochester, MN, United States

**Keywords:** gout, deep learning, dual-energy CT, time-efficiency study, segmentation

## Abstract

**Introduction:**

Dual-energy CT (DECT) is a non-invasive way to determine the presence of monosodium urate (MSU) crystals in the workup of gout. Color-coding distinguishes MSU from calcium following material decomposition and post-processing. Manually identifying these foci (most commonly labeled green) is tedious, and an automated detection system could streamline the process. This study aims to evaluate the impact of a deep-learning (DL) algorithm developed for detecting green pixelations on DECT on reader time, accuracy, and confidence.

**Methods:**

We collected a sample of positive and negative DECTs, reviewed twice—once with and once without the DL tool—with a 2-week washout period. An attending musculoskeletal radiologist and a fellow separately reviewed the cases, simulating clinical workflow. Metrics such as time taken, confidence in diagnosis, and the tool's helpfulness were recorded and statistically analyzed.

**Results:**

We included thirty DECTs from different patients. The DL tool significantly reduced the reading time for the trainee radiologist (*p* = 0.02), but not for the attending radiologist (*p* = 0.15). Diagnostic confidence remained unchanged for both (*p* = 0.45). However, the DL model identified tiny MSU deposits that led to a change in diagnosis in two cases for the in-training radiologist and one case for the attending radiologist. In 3/3 of these cases, the diagnosis was correct when using DL.

**Conclusions:**

The implementation of the developed DL model slightly reduced reading time for our less experienced reader and led to improved diagnostic accuracy. There was no statistically significant difference in diagnostic confidence when studies were interpreted without and with the DL model.

## Introduction

**Figure 1:**
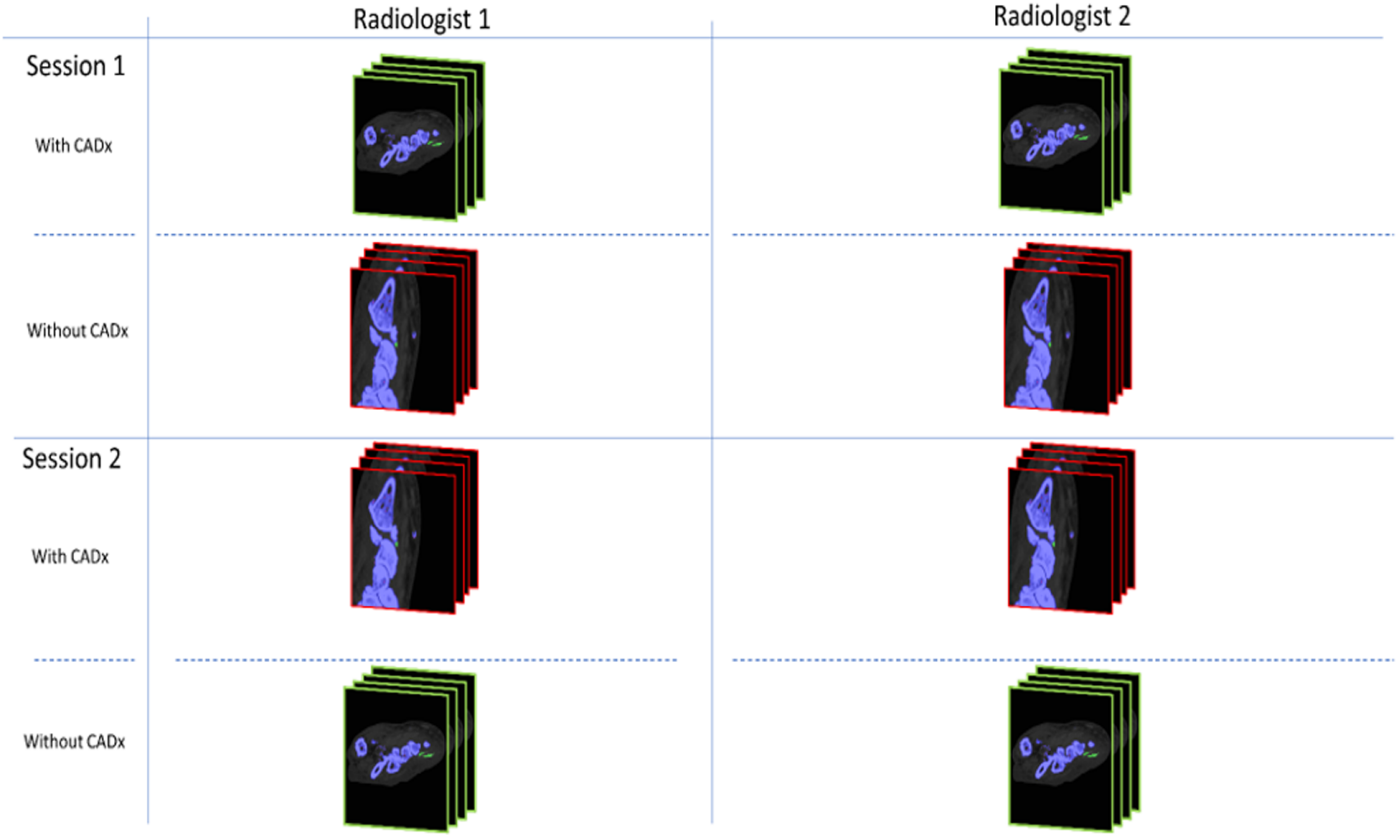
Schematic illustration of the review process. CADx, Computer assisted detection tool.

Dual-energy CT (DECT) is widely used in musculoskeletal radiology for various clinical purposes, such as diagnosing gout, pseudogout, inflammatory bone conditions, bone marrow tumors, and bone marrow edema (1–6) Specifically, DECT serves as a non-invasive method to determine the presence and burden of monosodium urate (MSU) crystals which plays a crucial role in the evaluation of gout (1). Through material decomposition and post-processing, pixels exhibiting x-ray attenuation consistent with MSU crystals are distinguished from calcium and are color-coded accordingly. In most software, MSU is labeled as green, while calcium is designated as blue (Figure 1). DECT exhibits high sensitivity and specificity in detecting MSU crystals (7, 8). A conclusive DECT result has been shown to reduce the need for confirmatory joint aspiration (1).

Recent advancements in AI can be attributed to deep learning (DL) technology, which is inspired by human neural networks (9, 10). In the field of radiology, computer-aided diagnosis (CAD) has been studied even before the emergence of DL (11). DL-based CAD has also been developed to detect lesions, such as brain hemorrhage on head CT (12), and pulmonary embolisms on chest radiographs (13). Evaluating the extent to which CAD can improve the performance of radiologists or raise the efficiency of the daily reading workflow is important. The usefulness of CAD, particularly for inexperienced radiologists, has been reported in the diagnosis of pulmonary nodules on CT images (14, 15).

Despite the diagnostic value of DECT in gout evaluation, the identification of small areas displaying green pixels within a large dataset can be a laborious diagnostic task. In cases with small amounts of green, the radiologist must carefully evaluate 3 planes of color-coded images to ensure that all pixels are identified and characterized appropriately. Automated detection using DL algorithms could enhance workflow efficiency. We hypothesized that a previously developed DL-based CAD with a mean Dice similarity coefficient (DSC) of 0.8934 would assist musculoskeletal radiologists in accurately assessing DECT during gout workup. This study sought to evaluate the impact of DL-based CAD on reading time, confidence, and diagnostic accuracy for the diagnosis of gout on DECT examinations, for both in-training and experienced radiologists.

## Methods

Institutional review board approval was obtained for this retrospective study. The requirement for informed consent was waived.

### Subjects

DECTs acquired between 7/23/2019 and 7/13/2022 were included in this study. Cases were identified from a pre-existing dataset of DECT examinations, with a final diagnosis of gout determined based on the criteria described in the 2015 Gout classification criteria: an American College of Rheumatology/European League Against Rheumatism collaborative initiative which uses imaging findings, joint aspiration, serum uric acid level, and/or clinical evaluation (16). The cohort of positive cases comprised consecutive participants with a positive diagnosis of gout. The cohort of negative cases comprised consecutive participants with a negative diagnosis of gout.

### Dual-energy CT images

All patients were scanned on a third-generation dual-source CT system (SOMATOM Force, Siemens Healthcare) with tube potentials of 80 kV(tube A) and 150 kV (tube B). Tin prefiltration was applied to the high-energy beam for improved spectral separation. The reconstructed images were analyzed using commercially available software (syngo.via VB30A, Siemens Healthcare). The software uses a material decomposition algorithm to identify uric acid and calcium voxels on the basis of their material-specific behavior under two different x-ray beam energy levels. No IV contrast material was used.

### Data preprocessing

Material decomposed DECT images were acquired in the axial (2-dimensional) plane and presented in a 3-color pixel image: green (MSU deposits), blue (calcium), and black (background). It is imperative to note that all DECT images in this study were processed using routine clinical protocols with no specialized modifications, ensuring consistency across all cases. This standard approach was applied uniformly for both human interpretation and Deep Learning-Computer Aided Detection (DL-CAD) systems. Using pydicom and nibabel packages (17, 18), the three-channel color-coded DECT images were transformed from 2-dimensional Digital Imaging and Communications in Medicine (DICOM) format to a three-channel, 3-dimensional format, Neuroimaging Informatics Technology Initiative (NIfTI), a segmentation-compatible format.

### Reading process

The experiment was designed as a prospective, randomized, crossover study, and it involved two separate review sessions for all cases. Two musculoskeletal radiologists participated in the study: an attending radiologist with 10 years of post-training subspecialty expertise in interpreting DECT scans (NR) and an in-training musculoskeletal radiology fellow (SP). All cases were reviewed twice, in two separate sessions, once with and once without the DL tool. A 2-week washout period was used between the two sessions to reduce reader-order bias and contextual bias (19, 20). During each session, half of the cases were randomly selected to be reviewed with the assistance of the CADx tool, while the other half were reviewed without the tool (Figure 1). This process allowed for fair comparison and evaluation of the DL tool's impact on the diagnostic process. The radiologists evaluated the post-processed DECTs to determine the presence or absence of gout. To simulate a real clinical workflow, they also distinguished false-positive green from MSU deposits.

The following data were recorded for each case during both review sessions: a. Time Taken: The time taken for the radiologists to evaluate each case. b. Diagnosis: Gout yes or no. c. Confidence Levels: The radiologists' confidence in their gout diagnosis was rated on a 5-point Likert scale (0–4). d. DL Tool's Helpfulness: The radiologists subjectively rated the DL tool's helpfulness on a 3-point Likert scale (0–2) for each case. Furthermore, the accuracy, sensitivity, and specificity were calculated for each reader, both with and without the utilization of the DL tool. The distribution of the reading times and confidence scores was checked for normality using the Shapiro-Wilk test. The reading times and confidence scores were analyzed by comparing the trials using the Wilcoxon signed-rank test.

### Model

We utilized a previously developed Unet-based DL segmentation model[Our previous work/under review]. This model generates masks for green foci in red, enhancing their visibility and facilitating their identification for the detection of them. It achieves a sensitivity and specificity of 98.72% and 99.98%, respectively, with a DSC of 0.9999 for background pixels, 0.7868 for green pixels, and an average DSC of 0.8934 for both types of pixels. As a computer-assisted detection tool, the model also generates a comma-separable variable file containing the coordinates of the identified matches in each plane, facilitating effortless navigation.

## Results

Thirty subjects who had a DECT exam were included in this study. The median age of subjects was 65 years (interquartile range = 18). The exams comprised 19 (63.33%) feet and ankles, 7 (23.33%) wrists and hands, 3 (10%) knees, and 1 (3.34%) elbow. Patient demographics are summarized in Table 1.

**Table 1:** Participant characteristics and gout status.

Subject characteristics	All subjects (*N* = 30)
Median age in years (IQR)	65 (18)
Age range in years	40–89
Female	8/30 (26.67%)
Male	22/30 (73.33%)
Gout status	
Positive	18/30 (60%)
Negative	12/30 (40%)

IQR, interquartile range.

The mean and standard deviation (SD) reading time for the in-training radiologist was 183 (SD = 32.36) seconds with the DL model and 190 (SD = 32.97) seconds without the model (*p*-value = 0.02), resulting in a 3.68% reduction in reading time. The mean reading time for the attending radiologist was 102 (SD = 38.75) seconds with the DL model and 106.84 (SD = 36.54) seconds without the model (*p*-value = 0.15), resulting in a 4.45% reduction in reading time. There was no statistically significant difference in diagnostic confidence between the two conditions for either radiologist. Table 2 summarizes the diagnostic confidences of readers with and without using CADx. The subjective assessment of the DL tool's helpfulness was high for both radiologists, with a mean score of 1.7 (SD = 0.58) for the attending and 1.2 (SD = 0.47) for the in-training radiologist.

**Table 2:** Summary of diagnostic confidence, and subjective helpfulness of a deep learning model for the diagnosis of gout using dual-energy CT.

	Diagnostic confidence with the (0–2 scale) [mean(SD)]	Diagnostic confidence without the model (0–2 scale) [mean(SD)]	Subjective assessment of model helpfulness (0–2 scale) [mean(SD)]
Attending musculoskeletal radiologist	3.74 (0.72)	3.8 (0.54)	1.7 (0.58)
In-training musculoskeletal radiologist	3.47 (0.82)	3.3 (0.96)	1.2 (0.47)

SD, standard deviation.

**Figure 2:**
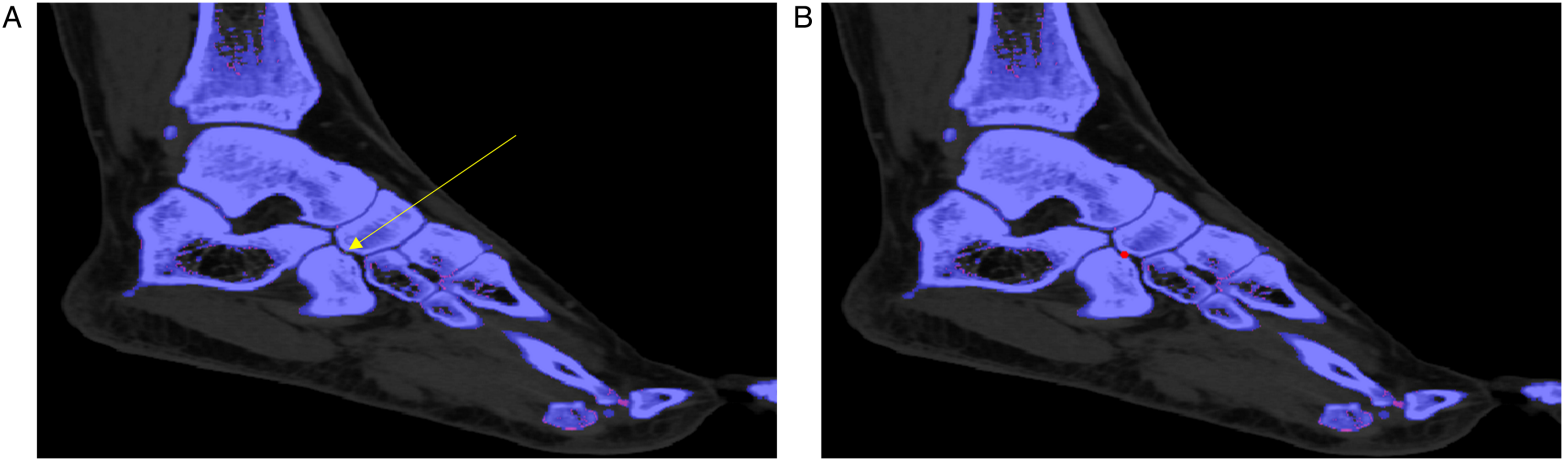
A sagittal view of the ankle dual-energy CT image of a patient diagnosed with gout. (**A**) The monosodium urate deposit coded in green, indicated by the yellow arrow at the cuboid-navicular joint space, was initially overlooked during the review process, but it was (**B**) detected using the deep learning tool as it makes the depositions stand out.

The diagnostic performances of the radiologists are summarized in Table 3. Although there were no statistically significant differences between the two occasions, the DL model identified tiny MSU deposits that led to a change in diagnosis in two cases for the in-training radiologist and one case for the attending radiologist (Figure 2). In 3/3 of these cases, the diagnosis was correct when using DL.

**Table 3:** Summary of diagnostic performance of the readers with and without using the deep learning tool.

	With deep learning tool			Without deep learning tool		
	Accuracy	Sensitivity	Specificity	Accuracy	Sensitivity	Specificity
Reader 1	86.67%	94.45%	75%	83.34%	88.89%	75%
Reader 2	93.34%	100%	83.34%	86.67%	88.89%	83.34%

## Discussion

The present study investigated the use of a DL algorithm to detect MSU deposits on DECT as a part of a gout workup. The results showed that the DL algorithm was able to reduce reading time for the in-training radiologist, without a significant impact on diagnostic confidence. Additionally, the DL algorithm identified tiny MSU deposits that led to an appropriate change in diagnosis in 5% of cases.

These findings suggest that even early DL algorithms have the potential to improve the efficiency and accuracy of gout diagnosis with DECT. This is particularly important for less experienced or in-training radiologists, who may not have as much experience with DECT interpretation and require more time to review the post-processed images. Although time saving was not significant for the attending radiologist, even incremental improvements in efficiency have the potential to make a busy work day more efficient. This current model scrutiny of all images. A trusted algorithm may lead to less time intensive review.

The ability to identify tiny MSU deposits could also be beneficial for patients with early gout or those with low disease burden, who may not have other obvious signs of the disease. In 3 cases, tiny deposits such as these were only noticed when utilizing the DL tool. The reviewing radiologist changed their diagnosis to “yes” in all 3 cases, which agreed with the reference standard. Identifying tiny deposits in characteristic locations may allow radiologists to make a more accurate diagnosis, helping patients receive appropriate treatment sooner and improve patient outcomes. The superior detection capability of DL-CAD can be attributed to its advanced pattern recognition algorithms, which can discern subtle anomalies that might elude human observation, particularly in cases of low disease burden or early-stage gout.

Thus far, DL models in musculoskeletal radiology have primarily focused on applying them to radiographs for tasks such as fracture detection, osteoarthritis grading, bone age assessment, quantification, and characterization of orthopedic implants (21–26). While fewer studies have been conducted on musculoskeletal MRI and CT, there are a growing number of investigations describing DL for image reconstruction, tissue segmentation, and detection of musculoskeletal diseases using cross-sectional imaging (21, 27–29). In the domain of disease detection using MRI scans, a majority of DL-based studies have been performed on the knee joint. These studies have concentrated on detecting various pathologies within the knee, including cruciate ligament tears, meniscus tears, and the integrity of the articular surface. Research in other joints and musculoskeletal regions has been limited. DL solutions have the potential to reduce the burden of the most time-intensive studies for radiologists.

Limitations of the current study include the small number of cases reviewed and the evaluation by only two readers. Further studies with larger sample sizes and larger reading cohorts are needed to confirm the findings of the present study and to assess the long-term clinical impact of using DL algorithms in gout diagnosis. Additionally, the DL algorithm was trained on a dataset of DECT images from the same institution.

Despite these limitations, the present study provides promising evidence for the use of DL algorithms in gout diagnosis with DECT. The present study suggests the future potential value of computer-aided diagnosis tools in detecting MSU deposits on DECT Further algorithm improvement and study is needed to confirm our findings and to establish the clinical impact of using DL algorithms in gout diagnosis.

## Data Availability

The datasets presented in this article are not readily available because; Data sharing should be run through our institution (Mayo Clinic) through IRB process. Requests to access the datasets should be directed to tiegsheiden.christin@mayo.edu.
